# Initial testing of pegfilgrastim (Neulasta Onpro) on‐body injector in multiple radiological imaging environments

**DOI:** 10.1002/acm2.13156

**Published:** 2021-01-04

**Authors:** Zaiyang Long, Anil Nicholas Kurup, Nicole M. Jensen, Nicholas J. Hangiandreou, Beth A. Schueler, Lifeng Yu, Shuai Leng, Christopher P. Wood, Joel P. Felmlee

**Affiliations:** ^1^ Department of Radiology Mayo Clinic Rochester MN USA

**Keywords:** CT, MRI, neulasta, pegfilgrastim, ultrasound, x‐ray

## Abstract

**Purpose:**

An increasing number of implantable or external devices can impact whether patients can receive radiological imaging examinations. This study examines and tests the Neulasta (pegfilgrastim) Onpro on‐body injector in multiple imaging environments.

**Methods:**

The injector was analyzed for four imaging modalities with testing protocols and strategies developed for each modality. In x‐ray and computed tomography (CT), scans with much higher exposure than clinical protocols were performed with the device attached to an anthropomorphic phantom. The device was monitored until the completion of drug delivery. For magnetic resonance imaging (MRI), the device was assessed using a hand‐held magnet and underwent the magnetically induced displacement testing in a 1.5T clinical MRI scanner room. For ultrasound, magnetic field changes were measured around an ultrasound scanner system with three transducers.

**Results:**

For x‐ray and CT no sign of device error was identified during or after the high radiation exposure scans. Drug delivery was completed at expected timing with expected volume. For MRI the device showed significant attractive force towards the hand‐held magnet and a 50‐degree deflection angle at 50 cm from the opening of the scanner bore. No further assessment from the gradient or radiofrequency field was deemed necessary. For ultrasound the maximum magnetic field change from baseline was measured to be +11.7 μT in comparison to +74.2 μT at 4 inches from a working microwave.

**Conclusions:**

No device performance issue was identified under the extreme test conditions in x‐ray or CT. The device was found to be MR Unsafe. Magnetic field changes around an ultrasound system met the limitation set by manufacture. Patient ultrasound scanning is considered safe as long as the transducers do not inadvertently loosen the device.

## 1 INTRODUCTION

Implanted and external/wearable devices providing medical care for patients could pose a range of risks in various imaging situations and consequently warrant careful investigation and decision making in daily radiology practice. For example, cardiac implantable electronic devices (CIED), that is, pacemaker and implantable cardioverter defibrillator systems, were extensively evaluated for modalities involving ionizing radiation, especially computed tomography (CT),^1^, ^2^, ^3^ and a non‐ionizing radiation modality magnetic resonance imaging (MRI).^4^ CIED issues such as oversensing, even though mostly being transient, were identified in the initial *in vitro* phantom studies at various exposure levels and clinical case reports.^1^, ^2^, ^5^ Hussein et al. retrospectively reviewed 516 patients with CIED undergoing clinically indicated CT examinations and did not find any primary clinically significant adverse events such as inappropriate shock or resetting of the devices or increased changes in device parameters.^6^ Based on these studies, various professional societies and experts in the field issued multiple sets of safety recommendations for CT and MRI imaging in patients with CIED.^7^, ^8^, ^9^ Furthermore, protocols and precautions required to safely complete an MRI examination of patients with certain CIED labeled as MR Unsafe by manufacturers have been reported.^10^, ^11^, ^12^, ^13^ Technical guidance has also been provided for devices other than CIED, such as neurostimulators, especially in the MRI environment.^9^, ^14^, ^15^

However, a rapidly growing number of new implantable or external devices bring new challenges to radiology practice because imaging exams have been labeled as contradicted for some of these devices. One such device is the Neulasta (pegfilgrastim) Onpro on‐body injector (Amgen Inc., Thousand Oaks, CA).^16^ It is designed to be applied onto patients’ skin of the abdomen or the upper arm via adhesive and subcutaneously deliver pegfilgrastim around 27 h after system activation, over a 45‐min period. Pegfilgrastim is a long‐acting recombinant human granulocyte colony‐stimulating factor analog prescribed to stimulate white blood cell production in patients at risk of febrile neutropenia after myelosuppressive chemotherapy. Pegfilgrastim must be administered 24 h after chemotherapy to minimize potential hematologic toxicity.^17^ Consequently, the on‐body injector was introduced in 2015 in an effort to save patients the time, infection exposure risk and costs associated with an additional clinic visit. The device use is increasing — now ~150 patients each month at our institution.

Concerningly, as FDA clearance of the injector delivery system did not require testing its safety in medical imaging environments, the manufacturer's instructions state not to expose this device to diagnostic imaging examinations “because the on‐body injector may be damaged and the patient could be injured” and specifically list x‐ray, CT, MRI, and ultrasound. A recent study investigated reasons why patients refused to receive this device, one of which was due to a scheduled MRI examination.^18^ However, some patients may not be aware of their upcoming imaging appointments or might need emergent scans before the completion of drug delivery. As a result, if this device is identified, these patients could be turned away from an important radiologic exam. Alternatively, the device might be removed prior to imaging, prompting a separate appointment for manual pegfilgrastim injection. No literature has investigated this device in the above‐mentioned multiple imaging modalities. Therefore, the aim of this work was to conduct a pilot study of the Neulasta Onpro device in these diagnostic imaging environments.

## 2 MATERIALS AND METHODS

**FIG. 1:**
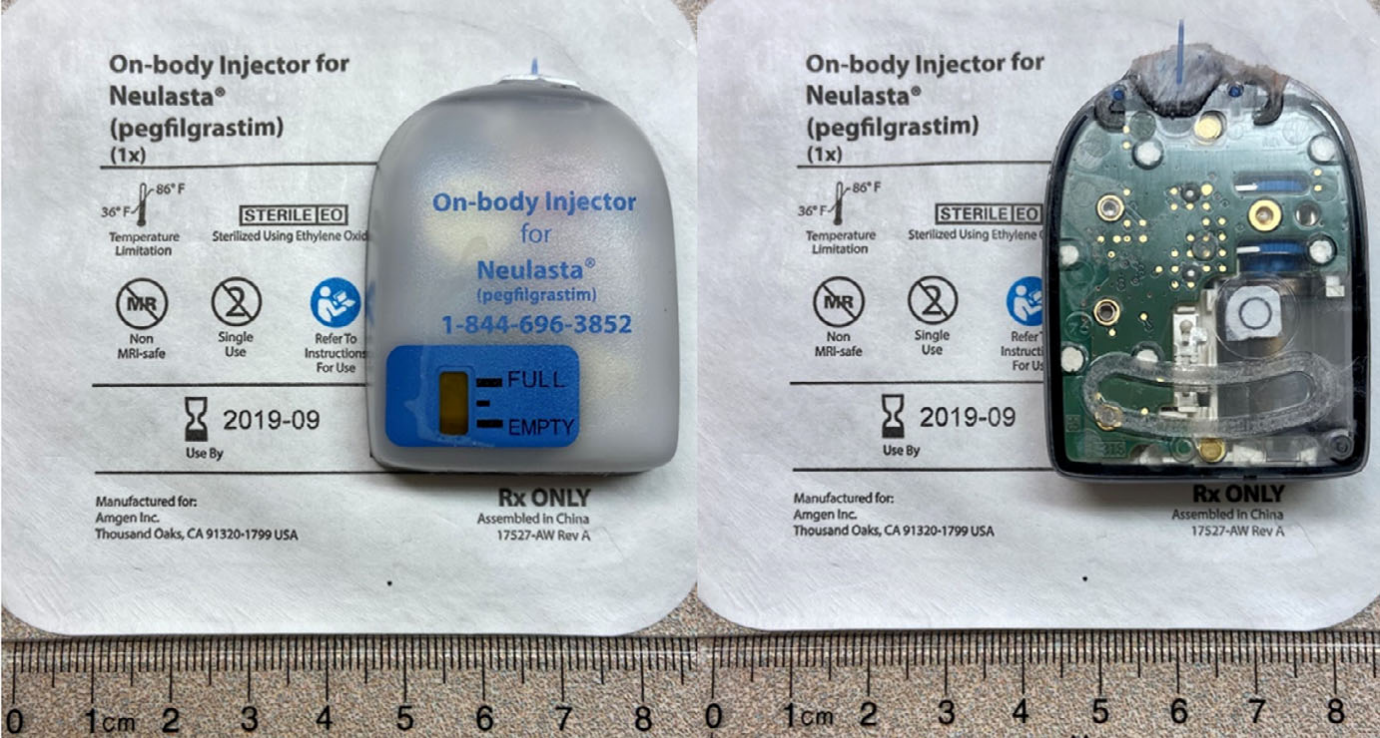
Photographs of a used Neulasta (pegfilgrastim) Onpro on‐body injector (left), as well as the components revealed by removing the adhesive from its back (right).

The on‐body injector components include a plastic housing, acrylic adhesive, electronic circuit board, batteries, reservoir, cannula introducer and cannula. Its size is approximately 5 cm × 3.8 cm × 1.4 cm as shown in Fig. 1. In the current investigations, the main risk of device malfunction was considered to be incorrect timing of drug delivery or incomplete dosing. A change in the delivery rate was a secondary concern, unless being very significantly changed (e.g., delivery over several hours). This device is designed to flash a red light and continuously beep for 5 min if it encounters an error, which assists with creating patient awareness of an error and in avoiding incorrect drug delivery.

An analysis was first performed of the on‐body injector for each imaging modality mentioned in the manufacturer's instructions. The interaction with ionizing radiation may cause free electrons in the electronic circuit which could form a small current or change in the voltage. However, in diagnostic x‐ray and CT examinations, the exposure levels associated with clinical exam protocols are very low. To stress the device, scans at exposure levels much higher than those in routine clinical practice were determined to be used to identify risks under these extreme conditions. For MRI, the risks associated with the main static magnetic field, gradient field, and radiofrequency (RF) field all require assessment for potential device displacement, malfunction, and/or heating issues. Finally, for ultrasound, direct exposure of the injector in the field of view is straightforward to avoid because the device is typically not in the plane of the transmitted/received ultrasound waves. If it is, that is, when scanning from the opposite side of the abdomen or arm area where the device is attached to, the attenuation and reflection of the ultrasound waves would be extremely high with the tissue layers, adhesive, plastic housing, and air gap in between that the influence on the device would be negligible. Therefore, no direct device testing for ultrasound was deemed necessary, and the potential electromagnetic interference was considered similar to other sources of electromagnetic interference as described below. The manufacturer instructs patients to keep the device at least 4 inches away from a microwave. Therefore, the magnetic field changes were measured around a GE Venue ultrasound scanner (GE Healthcare, Milwaukee, WI) while using a curvilinear, a linear and a sector transducer, which were further compared to field changes measured at 4 and 5 inches away from a working microwave, via the magnetometer function in the Physics Toolbox Sensor Suite application (Vieyra Software, Washington, DC) on an iPhone (Apple Inc., Cupertino, CA) without the presence of the device.

Based on the above analysis and magnetic field measurements, three on‐body injectors were purchased and each was tested for general x‐ray, CT, and MRI. Tests were performed as follows.

1. For CT testing, the device was prepared according to the manufacturer's instructions. The device was attached to a WYPALL general purpose wiper sheet (Kimberly‐Clark, Irving, TX) via its adhesive and made sure that the activation and cannula insertion had been correctly initiated. The device and the sheet were then taped on the upper abdominal area of a custom anthropomorphic phantom (The Phantom Laboratory, Salem, NY). The phantom was scanned on a 192 slice CT scanner (SOMATOM Force, Siemens Healthineers, Forchheim, Germany). Posterior–anterior (PA) and lateral tomograms were performed, followed by a series of ten scans using a high exposure abdomen‐based protocol without automatic exposure control (120 kV, 192 × 0.6 mm collimation with flying focal spot, effective mAs of 350, 0.5 s rotation time, pitch 0.6). The volume CT dose index (CTDIvol) for each of these scans was 23.4 mGy, resulting in a total of 234 mGy. CT scans covered a longitudinal range of 19 cm, with the device fully inside the scan range. The device was checked in the middle of the scans and afterwards for its built‐in error indicators and visible leakage. It was monitored for the entire time until the completion of the drug delivery process. The drug was collected into a container, and its volume was measured.
2. For general x‐ray testing, similar to the CT methods, the device was prepared and attached to 1‐cm thick foam. It was placed on the same anthropomorphic phantom. Scans were performed on a Philips PCR Eleva system (Philips Healthcare, Best, The Netherlands). A high exposure protocol was utilized and comprised of 10 exposures at 80 kV, each with 70 mGy entrance skin air kerma (EAK) over 6 s for a total EAK of 700 mGy. EAK was measured using a RaySafe X2 dosimeter system with the R/F sensor (Fluke Biomedical, Everest, WA). The device was also monitored until completion of the drug delivery.
3. For MRI testing, “MR Unsafe” was noted in the manufacturer's information (Fig. 1). First, the device was tested for ferromagnetic attraction with a Model 6860 doughnut hand‐held magnet (Boston Scientific Corporation, St. Paul, MN). Then the magnetically induced displacement test as described below was performed in Zone 4 of a 1.5T GE Optima MR450w MRI scanner (GE Healthcare, Milwaukee, WI).^19^ A piece of 20‐cm long fishing line, whose weight was <1% of the device (~27 g), was used to suspend the device from a holder apparatus with a plastic protractor. The whole system was placed on the patient gantry table and slowly advanced along the axis of the bore to 50 cm from the opening of the bore, where the spatial gradient is within 20% of the max spatial gradient along the axis of the bore. Considering that the injector is only attached to the patient by adhesive, if any significant displacement (i.e., projectile effect) was identified that would prevent the device from going into the bore, no further test would be performed to assess the impact of the time‐varying gradient field and the radiofrequency (RF) field. Otherwise, MRI scans would have been performed with the device to assess potential device malfunction or heating issues.

## 3 RESULTS

**FIG. 2:**
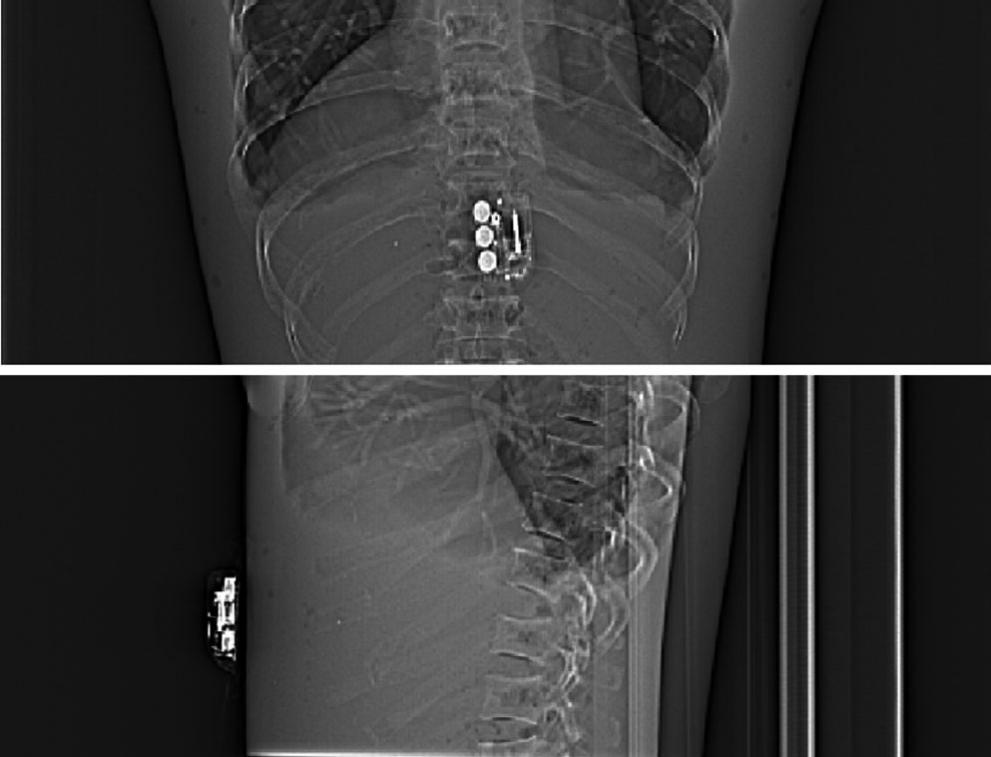
Posterior‐anterior and lateral computed tomography topogram images of the custom anthropomorphic phantom with the device placed on the upper abdomen.

**FIG. 3:**
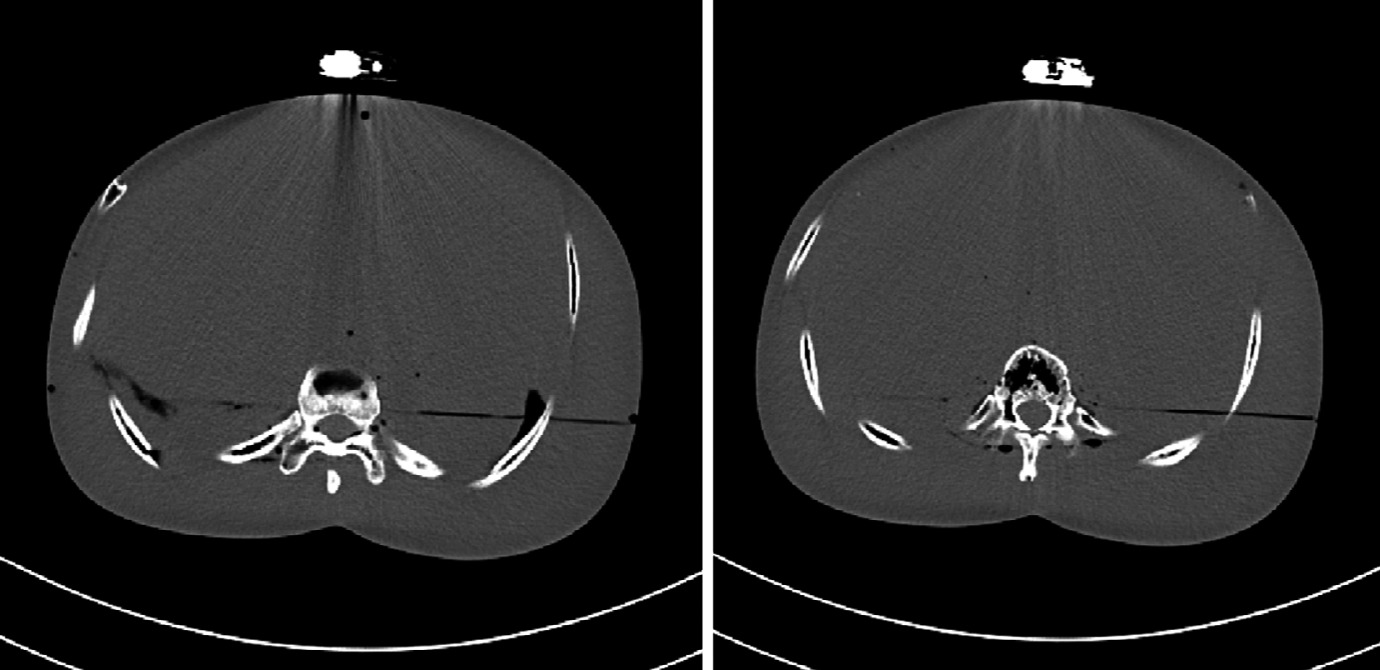
Example of two 5‐mm‐thick axial images reconstructed with a soft tissue kernel using a 400/40 HU window width/level setting, demonstrating the metal artifacts associated with the device.

**FIG. 4:**
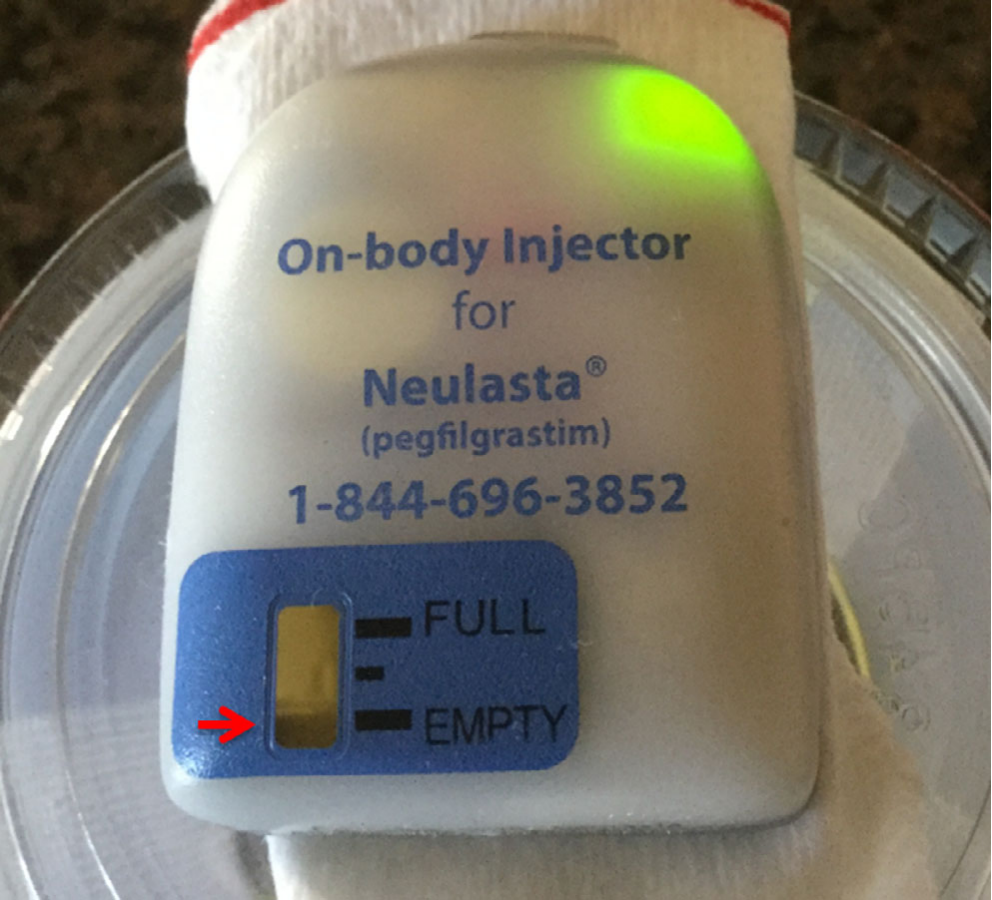
Photograph of the device at the end of drug delivery after ten computed tomography scans with a cumulative dose of 230 mGy CTDIvol. As expected, the indicator read empty (arrow), and the status light flashed green.

For device testing in CT, the device operation after exposure was found to be unaffected and matched the manufacturer's instructions, including the status of the fill indicator and status light through different stages. Figure 2 shows the PA and lateral topogram images which provided radiograph identification of the device. The device generated some metal artifacts which were demonstrated in two 5‐mm axial slices reconstructed with a soft tissue kernel (Fig. 3). No flashing red light, continuous beeping or visible leakage was observed during or after the ten scans. Starting around 26 h and 50 min, the device beeped and then after a 2‐minute time period, it started to deliver the medication. After another 45 min, it completed drug delivery with the expected fast flashing green light. The volume was measured to be 0.6 ml, and a black line on the fill indicator showed that the device was empty (Fig. 4).

**FIG. 5:**
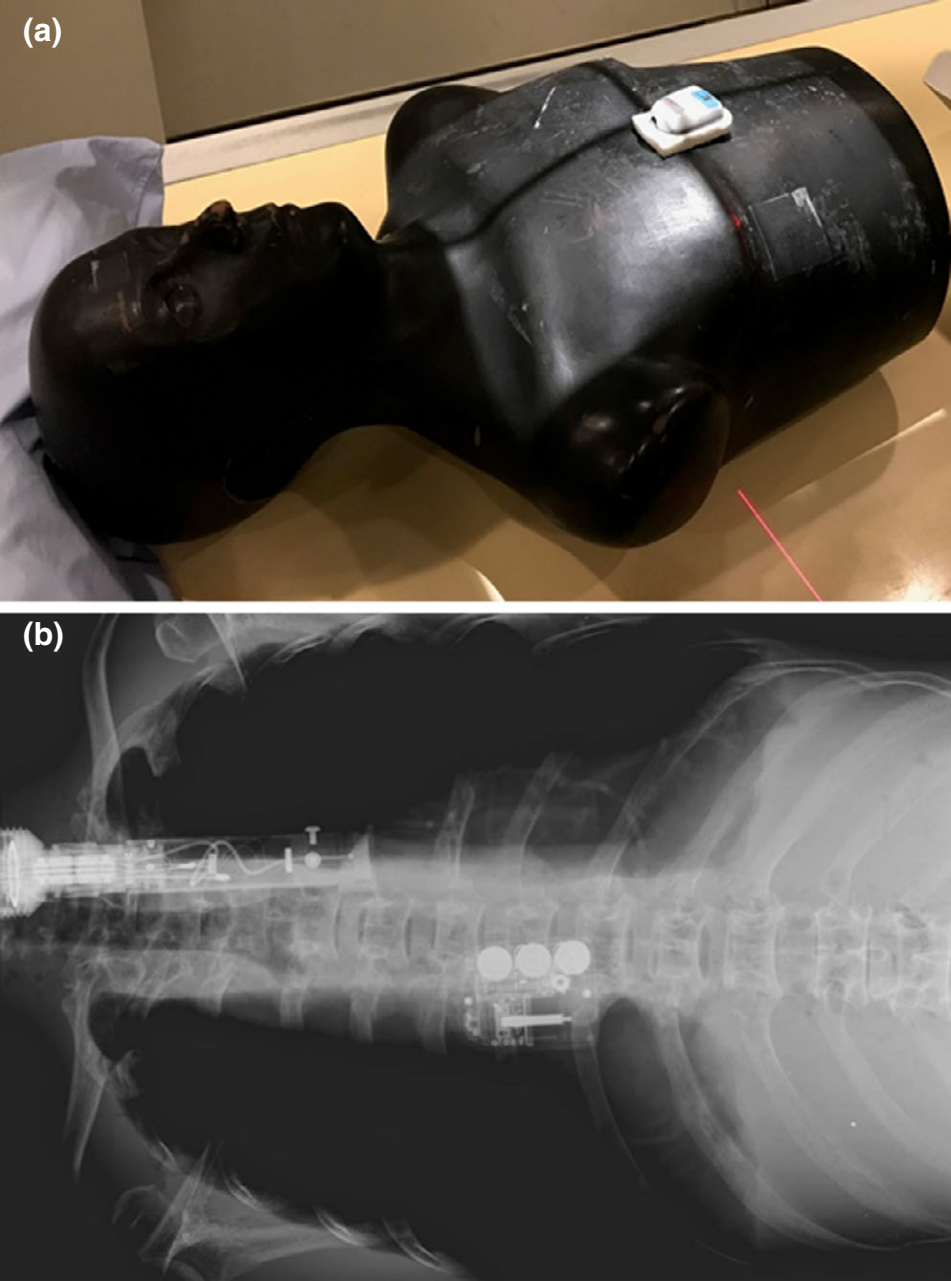
The experimental setup (a) and a radiograph (b) of the custom anthropomorphic phantom with the device in place for general x‐ray testing.

For general x‐ray testing, the experimental setup and a radiograph of the phantom with the device are shown in Fig. 5. The device operation was also found to be unaffected and no leakage was observed after the exposure. Finally, the drug was delivered at the expected time and with no change in the expected rate or volume.

**FIG. 6:**
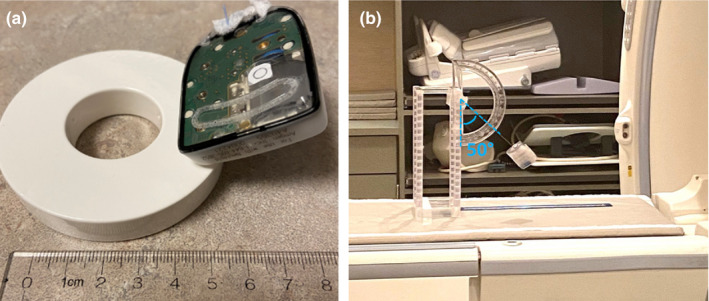
(a) Photograph of the portion of the device that showed the strongest pull force toward the hand‐held magnet. (b) The magnetically induced displacement test in Zone 4 of a 1.5T GE MRI scanner showed a deflection angle of 50 degrees.

For MRI testing, the device demonstrated an obvious attractive force using the hand‐held magnet. The portion of the device that generated the strongest pull force can be seen in Fig. 6(a), as demonstrated with an opened device with the electronic circuit board revealed. Furthermore, the device showed a deflection angle of 50 degrees at 50 cm from the opening of the bore [Fig. 6(b)], corresponding to a magnetically induced displacement force of Fm = mg × tan (50°) = 0.3 N. The magnetic field at this location was 131.9 mT, and the spatial gradient of the magnetic field was 5.2 mT/cm.

For the ultrasound evaluation, the magnetic field changes were within −5.8 to +3.5 μT from baseline at 5 inches away from the ultrasound scanner and transducers. A maximum change of +11.7 μT was observed when the meter came into contact with the curvilinear transducer, which was still lower than a maximum change of +19.5 μT at 5 inches and +74.2 μT at 4 inches away from a working microwave. No further test with a device in the ultrasound environment was deemed necessary.

## 4 DISCUSSION

In this pilot study, safety testing of the Neulasta Onpro on‐body injector was performed in the diagnostic x‐ray, CT, MRI and ultrasound imaging environments. Exposure levels used for the x‐ray and CT testing were purposefully selected to be very high with the device directly in the field of view, in order to determine any significant adverse event under these extreme conditions (~400 times of the EAK from an average‐sized adult lateral lumbar spine radiograph for x‐ray testing, and ~20 times of the CTDIvol from an average‐sized adult abdominal scan for CT testing). Nevertheless, the device operated correctly and completed drug delivery at the expected timing with the expected volume and delivery rate. No sign of error was observed. Metal artifacts were noted in the CT images using a soft tissue display window setting. Metal implants are frequently seen in CT examinations today and many phantom and patient studies have evaluated the efficacy of various metal artifact reduction algorithms.^20^, ^21^, ^22^, ^23^ The artifacts caused by this device were less severe in appearance compared to other common implants such as dental or orthopedic implants. But the clinical relevance could be further evaluated and a metal artifact reduction algorithm could be utilized to reduce the artifacts.

In a clinical 1.5T MRI scanner room, this device generated an attractive magnetic force larger than the gravitational force on the device at 50 cm from the scanner bore. Its own adhesive cannot safely prevent the projectile effect and no accessory should be applied to hold the device in place. Therefore, the device was labeled as MR Unsafe in our practice and the results prompted inclusion on the patient MRI screening form to aid in the identification of patients with these devices. For ultrasound, electromagnetic interference with the device would be extremely unlikely as the magnetic field changes observed around an ultrasound scanner and transducers were very low, within the earth's natural magnetic field range and lower than the range from a working microwave as provided by the manufacturer. Direct physical contact of the device with the transducer and coupling gel may unintentionally affect the adhesive or loosening the device. Therefore, it was deemed to be safe to perform an ultrasound scan as long as direct physical contact of the transducer and coupling gel with the device can be avoided.

When considering these types of devices in Radiology practice, patient anatomy, physiology, and potential disease status also play a significant role. For example, the potential cost associated with patient hospitalization due to a missed dose of medication should be weighed in the patient management decision.^24^ An additional factor is whether the device has a clear indicator of error and therefore can be easily monitored. The on‐body injector device has both visual and audible error indicators. Normal device failure rate should also be accounted for when interpreting these results. Even though no failure was observed in our pilot test, the device's baseline failure rate in patients not exposed to imaging environments has been reported to be up to 7%, including delivery failure or device leakage.^25^, ^26^

Many implantable and external devices are considered contraindications for imaging exams by their manufacturers.^27^ Blanket statements of contradiction to medical imaging without testing are expeditious and potentially limit manufacturer liability, but they could negatively impact patient care. Diagnostic imaging is essential to the care of most patients, particularly those with major comorbidities who often also require such implanted and external devices. While some devices are indeed unsafe or pose significant risks while being close to imaging equipment or being scanned, we strongly recommend that manufacturers perform thorough tests of their devices in routine medical imaging environments and use data‐supported recommendations for patient care. Alternatively, regulatory bodies should consider requiring evaluation of the effects of medical imaging environments on such devices, including implanted medication delivery systems. In the absence of adequate testing by manufacturers prior to FDA clearance, necessary imaging may be postponed, radiology departments and providers faced with clinical scenarios that prompt imaging may develop haphazard policies to deal with devices, and institutions expends significant effort to flag patients with devices for appropriate scheduling of their imaging exams and avoid inadvertent imaging of devices with contraindications.

There are a number of limitations in this pilot study. First, the number of the tested injectors was very limited due to cost constraints and only three devices were purchased for testing. Furthermore, test conditions were limited for the same reason. Only very high exposure levels were tested for x‐ray and CT and used to identify risks associated with worst‐case conditions. Future study could be more comprehensive using various exposure levels in a controlled fashion. However, results presented indicate no impact on device operation by general x‐ray, CT, or ultrasound examinations along with verification that the device is unsafe in MRI. This information could be used to develop in‐depth tests and processes to guide patient management, especially for patients with urgent imaging need or being inadvertently scanned. The described consideration process and testing methods could also be applied to other new devices.

## 5 CONCLUSION

The Neulasta Onpro on‐body injector was investigated in diagnostic x‐ray, CT, MRI, and ultrasound environments. Under the extreme test conditions, the device functioned as expected in x‐ray and CT. The device is MR Unsafe. Ultrasound examinations are safe to perform as long as cares are taken to avoid loosening the device inadvertently.

## AUTHOR CONTRIBUTION

Zaiyang Long – study design and execution, data analysis, manuscript preparation, manuscript critical review, and approval; Anil Nicholas Kurup – study discussion, clinical inputs, manuscript preparation, manuscript critical review, and approval; Nicole M Jensen – clinical inputs, manuscript critical review, and approval; Nicholas J Hangiandreou – study design, discussion, manuscript critical review, and approval; Beth A Schueler – study design and execution, discussion, manuscript critical review, and approval; Lifeng Yu – study design and execution, discussion, manuscript critical review, and approval; Shuai Leng – study design, discussion, manuscript preparation, manuscript critical review, and approval; Christopher P Wood – study discussion, clinical inputs, manuscript preparation, manuscript critical review, and approval; Joel P Felmlee – study design and execution, discussion, manuscript preparation, manuscript critical review, and approval.
